# Mechanical Bowel Preparation Is a Risk Factor for Postoperative Delirium as It Alters the Gut Microbiota Composition: A Prospective Randomized Single-Center Study

**DOI:** 10.3389/fnagi.2022.847610

**Published:** 2022-04-04

**Authors:** Zhoujing Yang, Chuandi Tong, Xinye Qian, Hailian Wang, Yingwei Wang

**Affiliations:** Department of Anesthesiology, Huashan Hospital, Fudan University, Shanghai, China

**Keywords:** mechanical bowel preparation (MBP), gut microbiota, postoperative delirium (POD), gastrectomy, perioperative interventions

## Abstract

**Background and Objective:**

Postoperative delirium (POD) is a frequent complication in patients undergoing gastrectomy. Increasing evidence suggests that abnormal gut microbiota composition may contribute to its morbidity. However, it is unclear whether mechanical bowel preparation would cause postoperative delirium by altering the gut microbiota of patients. This study aimed to investigate the association between mechanical bowel preparation and postoperative delirium in patients undergoing gastrectomy.

**Methods:**

A prospective randomized single-center study was performed. A total of 81 patients with gastric cancer were enrolled and randomly assigned to two groups: preparation group and non-preparation group according to whether the patient received MBP before surgery. To diagnose postoperative delirium, we used the 3-Min Diagnostic Interview for Confusion Assessment Method-defined delirium for five successive days after surgery. 16s rRNA gene sequencing was used to investigate changes in the intestinal bacteria. The linear discriminant analysis and effect size (LefSe) analysis were also used to identify the different taxa of fecal microbiota between the postoperative delirium and non-postoperative delirium groups.

**Results:**

We found that there was a significant difference in β-diversity of the gut microbiota between the preparation group and non-preparation group (*P* = 0.048). Furthermore, patients in the preparation group had a much higher rate of postoperative delirium (13/40, 32.5%) compared with that in non-preparation groups (4/41, 9.8%). Multivariate regression analysis adjusted by other risk factors indicated that mechanical bowel preparation was associated with the occurrence of delirium (odds ratio = 4.792; 95% confidence interval: 1.274–18.028; *P* = 0.020). When comparing the gut microbiota of patients with and without POD, *Bacteroides* and *Veillonella* (genus), which were higher in the preparation group, were also higher in delirium patients (*P* < 0.05). Genus *Olsenella* was both relatively higher in the non-preparation group and non-POD group (*P* < 0.05).

**Conclusion:**

Mechanical bowel preparation not only altered the gut microbiota composition of patients with gastric cancer but also increased the incidence of postoperative delirium. Among all the gut microbiota altered by mechanical bowel preparation, *Bacteroides* and *Veillonella* genus might be a risk factor of POD. Genus *Olsenella* might be a beneficial bacteria to reduce the incidence of POD.

## Introduction

Postoperative delirium (POD) is a common complication after the operation, characterized by four features: an acute change in mental status with a fluctuating course, inattention, disorganized thinking, and an altered level of consciousness (Marcantonio, 2017; Oh et al., 2017). POD is associated with functional decline in normal activity, increased length of hospital days, higher costs, and other complications (Sprung et al., 2017). Its incidence ranges from 10 to 60% in patients receiving major abdominal surgery as reported (Brouquet et al., 2010). Various factors, namely, perioperative interventions and surgical procedures, are associated with POD.

Recent studies show that the gut microbiome can modulate brain function through the gut-brain axis, which is a complex bidirectional signaling system between the gut and the brain (Ridaura and Belkaid, 2015; Pascale et al., 2018). Moreover, studies are showing that abnormal gut microbiota composition after abdominal surgery may contribute to the pathogenesis of POD in mice (Zhang et al., 2019).

Mechanical bowel preparation (MBP) for elective gastrectomy is often used before abdominal surgeries. Previous studies focusing on the effects of mechanical bowel preparation on the intestinal microbiota are not consistent (Mai et al., 2006). Several studies have indicated that MBP did not have a significant impact on gut microbiota and it did not alter the microbial diversity even when the total bacterial load was halved (O’Brien et al., 2013). But other studies have found that bowel preparation had a substantial effect on the gut microbiota, and it might take 14 days for the majority of the intestinal microbiota to recover to the baseline composition (Nagata et al., 2019).

Thus, we conducted a single-center prospective randomized controlled study to verify our hypothesis that mechanical bowel preparations might be a risk factor of POD as MBP may change the state and composition of the gut microbiota in patients with gastric cancer.

## Materials and Methods

### Patient Enrollment and Ethics

A prospective randomized single-center study was conducted between November 2018 and November 2019 at the Huashan Hospital, Fudan University. The clinical trial was approved by the ethical committee of Huashan Hospital (approval number: KY2018-354) under the declaration of Helsinki. Every patient enrolled should sign the informed consent. This study was registered at http://www.chictr.org.cn/index.aspx (registration number: ChiCTR1800019139). The initial date of registration was 26/10/2018.

### Inclusion/Exclusion Criteria

Inclusion criteria: (1) patients were aged over 65 years old, no gender preference; (2) patient’s ASA grade (American Society of Anesthesiologists physical status): I–III; (3) patients were diagnosed with gastric cancer (T1M0N0 and T2M0N0) and were scheduled to undergo elective radical gastrectomy; and (4) patients were able to communicate with researchers without difficulty and follow all the protocol of the trial.

Patients with the following conditions were excluded: (1) history of neurological disease, dementia, and other psychiatric illness; (2) history of using probiotics, antibiotics, prebiotics, or synbiotics within 3 months before fecal sample collection; (3) preoperative Mini-Mental State Examination (MMSE) score less than 24; (4) history of severe auditory, visual, or motor deficits; (5) history of digestive system diseases other than cancer; (6) history of other serious primary diseases; (7) illiteracy or communicative disorders; and (8) recent participation in other clinical trials.

### Allocations

The patients were randomly assigned into two groups: the preparation group and the non-preparation group according to whether the patient received MBP before surgery. All participants enrolled in the study followed a recommended balanced diet during the study period (Drago et al., 2016).

Simple random treatment allocations were generated before starting the study and concealed in sequentially numbered and sealed opaque envelopes. After written informed consent was obtained, a patient was randomized by opening the next number envelope (Luangchosiri et al., 2015). An entire clinical trial of patients was performed by assigned anesthesiologists and surgeons who were not involved in the study and were blinded to the grouping.

Patients in the preparation group received a standard high-volume (2–4 L) polyethylene glycol electrolyte lavage solution (generic name: Polyethylene Glycol Electrolytes Powder, manufacturer: Shenzhen Wanhe Pharmaceutical Co., Ltd., SFDA approval number: H20030827) the day before surgery for mechanical bowel preparation, while patients in the non-preparation group did not receive the lavage solution (Drago et al., 2016). The administration could be terminated when the stool was clear, and the total amount of the lavage solution was not more than 4 L. Fecal samples of both the groups were collected in fecal collection containers twice, respectively. The first fecal samples were collected 2 days before surgery, while the second fecal samples were collected on the morning of the operation day. The containers were immediately stored in −80°C refrigerator (Jiang et al., 2019).

### Anesthesia Management

When entering the operation room, every patient received the same anesthesia protocol. Routine monitoring consisted of continuous electrocardiogram, pulse oximetry, non-invasive blood pressure, and end-tidal carbon dioxide monitoring. A Bispectral index monitoring (A-2000; Aspect Medical System, Newton, MA, United States) was applied to the forehead of the patient before the induction of anesthesia. The arterial catheter was also inserted before induction for continuous invasive arterial blood pressure measurements (Yi et al., 2020). Induction was performed using sufentanil 0.5–1 μg/kg, midazolam 1 mg, propofol 1.5–2 mg/kg, and cisatracurium 0.2 mg/kg. After tracheal intubation, mechanical ventilation with 60% oxygen was provided. Tidal volume was adjusted to maintain normal arterial carbon dioxide according to blood gas analysis. The depth of anesthesia was controlled by altering the inhaled sevoflurane concentration, based on the hemodynamic response and bispectral index (BIS) values (target values range from 40 to 60). The maintenance infusion rate of cisatracurium was 1–1.5 μg/kg/min. Sufentanil at a total dose of 2–3 μg/kg was administered during the surgery. If MABP < 70 mmHg, patients were treated with norepinephrine, or phenylephrine immediately to ensure their MABP was larger than 70 mmHg. Cardiovascular active drugs such as isoprotereno were used if HR < 50 bmp (anticholinergic drugs were avoided). Cefazolin was used 30–60 min before surgery to prevent infection (Maekawa et al., 2020). Cefazolin was added every 3 h or when the bleeding volume was greater than 1,000 ml. The temperature of patients was controlled and monitored at 36.3–36.9°C in our study. All patients were scheduled to undergo radical gastrectomy by the assigned four surgeons.

After surgery, short-acting analgesics, such as IV injection of morphine 5–10 mg, were required according to the numerical rating scale of the patient. The dose was adjusted according to individual conditions until sufficient analgesic effect is achieved.

### Basic Information Analysis

Demographic, anesthetic, and surgical information of all patients were documented to detect any statistical difference between the preparation and non-preparation groups.

The baseline cognitive function of the patients was assessed using the MMSE (score range 0–30) (Cryan and Dinan, 2012) before surgery, which was conducted by a physician of neurology who was blinded to the group assignment.

Postoperative recovery profiles and postoperative complications were also documented (Shin et al., 2015).

### Outcome Measures

#### Incidence of Postoperative Delirium

The primary outcome was the incidence of the POD. POD was diagnosed by the 3-Min Diagnostic Interview for Confusion Assessment Method-defined delirium (3D-CAM) (Inouye et al., 1990). It consisted of four criteria: (I) acute fluctuating mentation, inattention (II), disorganized thinking (III), and (IV) altered level of consciousness. Patients that met criteria I and II and either III or IV were diagnosed with postoperative delirium. Every patient was assessed twice a day (8:am–10:am and 4:00 pm–6:00 pm) for 5 days successively after surgery by 2 trained physicians blinded to the trial.

#### Severity of Postoperative Delirium

For patients diagnosed with POD, the severity of delirium was then assessed by the short form of the Confusion Assessment Method-Severity (CAM-S). The assessment lasted until the score of patients of 3D-CAM returned to normal (Mutch et al., 2018). We recorded each score of CAM-S short form of patients diagnosed with POD and the duration of POD. Also, patients were evaluated by 2 trained physicians blinded to the trial.

#### Difference of Gut Microbiota Composition Between the Preparation Group and the Non-preparation Group

The 16S rDNA high-throughput sequencing was performed on fecal samples by Realbio Genomics Institute (Shanghai, China) to evaluate differences in the gut microbiota composition between the two groups. Bacterial diversity was assessed by α-diversity (Chao 1) and β diversity (principal coordinates analysis, PCoA) (Cole et al., 2014). The linear discriminant analysis (LDA) and effect size (LefSe) analysis were used to search different taxa of fecal microbiota between the two groups (Ren et al., 2020).

#### Basic Information and Alteration in the Taxa Between the Postoperative Delirium Groups and Non-postoperative Delirium Groups

Patients were diagnosed with delirium according to the 3D-CAM and thus all of them were assigned to two groups—the POD group and the non-POD group.

We documented demographic data, anesthetic and surgical data, and postoperative data in the POD group and non-POD group, analyzed the *P*-value, and conducted univariable and multivariate logistic regression to explore the risk factors of POD.

Apart from these, we examined the different microbiota between the second fecal samples of patients of the POD group and the non-POD group. The LefSe analysis was used to identify the different taxa of fecal microbiota between the POD and non-POD groups.

### Statistic Methods

The SPSS (ver. 21.0, SPSS Inc., Chicago, IL, United States) and R software (ver. 3.1.0, the R Project for Statistical Computing) were used for statistical analysis. In this study, all statistical tests were two-sided, and difference achieving values of *P* < 0.05 were considered statistically significant (Minai et al., 2014). We summarized patient demographics along with the surgical and anesthetic characteristics in the two groups. Data were expressed as mean [interquartile range (IQR) (range)] or mean (SD) and number (proportion). Categorical variables were compared by Pearson’s chi-squared test with a continuity correction, or Fisher’s exact test, if applicable (Minai et al., 2014). The Kolmogorov–Smirnov test was used to examine the normality of quantitative variables (Brons et al., 2012). Non-normally distributed quantitative variables were analyzed by the Mann–Whitney *U*-test (Minai et al., 2014). Normally distributed variables were analyzed by the Student’s *t*-test.

The incidence of POD between groups was analyzed by Fisher’s precision probability test. The severity and duration of POD between groups were analyzed by Mann–Whitney *U*-test. Baseline patient characteristics and MBP that were significant in the univariable analysis at a threshold *P* < 0.1 were entered into a forward multivariable logistic regression model. A multivariate logistic regression analysis was performed to evaluate the effects of the MBP after adjustment for potential confounding factors. *P*-values < 0.05 were considered to indicate statistical significance (Velayati et al., 2020).

All reads of fecal samples were deposited and grouped into operational taxonomic units (OUTs) at a sequence identity of 97%, and the taxonomic affiliation of the OTUs was determined according to quantitative insights into microbial ecology (QIME, version 2.0). The following downstream data analyses were conducted in R software. Bacterial diversity was determined by α diversity (Chao 1) and β diversity (PCoA). LEfSe was used as a tool to identify the differences in bacteria taxa between groups based on *P* < 0.05 and LDA score > 2.0 (Li et al., 2020).

The sample size was calculated based on the incidence of POD. The pilot trial showed that the incidence of POD was 30% (3/10) in the preparation group and 10% (1/10) in the control group, respectively. Assuming a two-sided α = 0.05 and statistical power of 0.8, the sample size was calculated to be 40 in each group. Considering a 20% loss to follow-up, we determined to enroll 96 patients in this study.

## Results

### Basic Information

**FIGURE 1:**
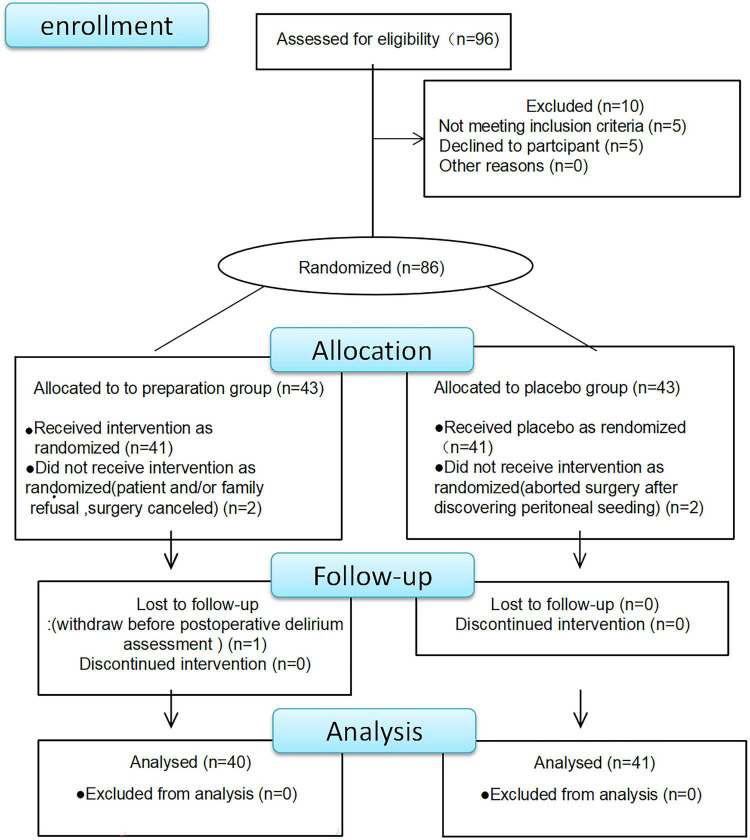
Participants’ recruitment and reasons for exclusions and treatment allocations.

A total of 96 patients were enrolled between November 2018 and November 2019. Figure 1 illustrated participant recruitment, reasons for exclusions, and treatment allocations. Of all the patients, 10 were excluded before the trial. Of the remaining 86 patients, three in the preparation group and two in the non-preparation group dropped out. Finally, 40 subjects in the preparation group and 41 subjects in the non-preparation group were eligible for this trial.

Table 1 illustrated the epidemiological information of all the patients recruited. There was no significant difference between the two groups in demographic information (Table 1). Detailed data of anesthesia and surgery were presented in Table 2. There was no significant between the two groups. As shown in Table 3, postoperative pain scores at 24, 48, and 72 h, and total morphine consumption were also similar between the two groups. There was also no significant difference in other clinical outcomes and postoperative complications between the two groups (Table 3).

**TABLE 1:** Demographic data in the preparation group and non-preparation group.

	Pre group (*n* = 40)	Non-pre group (*n* = 41)
Male sex; *n*	25 (62.5%)	22 (53.7%)
Age;yr	73 (5)	74 (4)
BMI; kg/m^2^	23.3 (3.0)	24.2 (3.1)
**Education**		
Primary school education	16 (40%)	21 (51%)
Secondary school education	21 (53%)	17 (42%)
University education	3 (7%)	3 (7%)
Heavy drinker*^1^		
Yes	10 (25%)	12 (29%)
No	30 (75%)	29 (71%)
**Current smoker**		
Yes	11 (28%)	9 (22%)
No	29 (72%)	32 (78%)
**ASA physical status**		
I	16 (40%)	15 (37%)
II	20 (50%)	21 (51%)
III	4 (10%)	5 (12%)
Preoperative baseline MMSE scores (0–30)	27.4 (1.6)	27.6(1.0)
Preoperative HAMD scores	2.6 (2.5)	3.0 (2.2)
**Hemoglobin**		
Normal	31 (78%)	33 (80%)
Abnormal	9 (22%)	8 (20%)
**Tumor stage**		
T1N0M0	12 (30%)	15 (37%)
T2N0M0	28 (70%)	26 (63%)
**Diabetes**		
Yes	13 (32.5%)	9 (22.0%)
No	27 (67.5%)	32 (78.0%)
Nutritional impairment*^2^		
Yes	2 (5%)	1 (2%)
No	38 (95%)	40 (98%)
Functional dependency*^3^		
Yes	0 (0%)	0 (0%)
No	40 (100%)	41 (100%)

Data were expressed as mean (SD), median [IQR (range)], or number (proportion)*. BMI, body mass index; MMSE, Mini-Mental State Examination; HAMD, Hamilton depression scale; pre group, preparation group; non-pre group, non-preparation group; *^1^Defined as current intake of alcohol, on average, 3–4 drinks per day at least four times per week; *^2^Defined as BMI < 18.5 kg/m^2^; *^3^Defined as Functional Activities Questionnaire (FAQ) score ≥ 5.

**TABLE 2:** Anesthetic and surgical data in the preparation and non-preparation groups.

	Pre group (*n* = 25)	Non-pre group (*n* = 26)	*P*-value
Duration of surgery; min	190 [158–229 (95–436)]	210 [158–250 (120–407)]	0.431
Duration of anesthesia; min	250 [206–276 (130–480)]	255 [213–300 (155–466)]	0.422
EBL; ml	150 [100–200 (50–600)]	150 [100–275 (40–500)]	0.269

*Data were expressed as mean (SD), median [IQR (range)], or number (proportion). EBL, estimated blood loss; pre group, preparation group; non-pre group, non-preparation group.*

**TABLE 3:** Postoperative data for the preparation group and non-preparation groups.

	Pre group (*n* = 40)	Non-pre group (*n* = 41)	*P*-value
**Pain NRS score (0–10)**			
24 h	3 [3–5 (0–9)]	3 [2–4 (0–7)]	0.346
48 h	3 [2–3 (0–5)]	3 [2–3 (0–5)]	0.335
72 h	2 [0–3 (0–3)]	1 [0–2 (0–3)]	0.274
Cumulative rescue morphine consumption; mg	4.3 (1.8)	4.7 (2.1)	0.225
Postoperative time out of bed; days	3 [2–4 (1–6)]	3 [2–4 (1–7)]	0.285
Length of hospital stay; days	8 [7–10 (3–21)]	9 [7–11 (5–24)]	0.337
**Postoperative complications:**			
Wound infection rate	2/40 (5.0%)	1/41 (2.4%)	0.616
Anastomotic leak rate	1/40 (2.5%)	0/41 (0.0%)	0.494
Postoperative bleeding rate	0/40 (0.0%)	1/41 (2.4%)	0.999
Gastroparesis rate	0/40 (0.0%)	0/41 (0.0%)	0.999
Dumping Syndrome rate	0/40 (0.0%)	0/41 (0.0%)	0.999
Duodenal stump rupture rate	0/40 (0.0%)	0/41 (0.0%)	0.999
Intestinal obstruction rate	1/40 (2.5%)	1/41 (2.4%)	0.999

*Data were expressed as median (IQR[range]) or mean (SD) and number (proportion). NRS, numerical rating scale; pre group, preparation group; non-pre group, non-preparation group.*

### Mechanical Bowel Preparation and Postoperative Delirium

**FIGURE 2:**
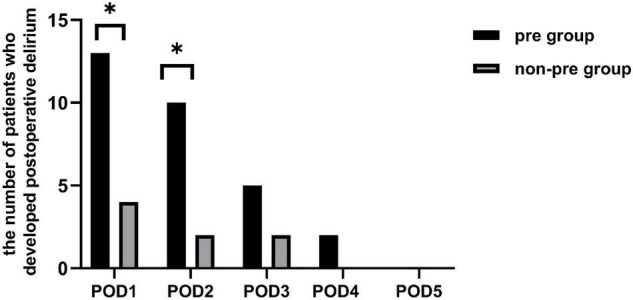
The number of patients who developed postoperative delirium. There existed a difference between the two groups in the individual prevalence of delirium on POD 1 and 2 (*P* = 0.025 and *P* = 0.025). There was no significant difference between the two groups in the individual prevalence of delirium on POD 3, 4, and 5 (*P* = 0.409, *P* = 0.241, and *P* = 0.999, respectively). *pre-group*, *preparation group; non-pre group, non-preparation group.* *P < 0.05.

Of all the 81 patients, 17 developed POD, making its morbidity 21.0%. The incidence of postoperative delirium was significantly higher in the preparation group than in the non-preparation group [32.5% (13 of 40) vs. 9.8% (4 of 41), *P* = 0.025]. Figure 2 showed the number of patients with delirium on each of the 5 days after surgery from both groups. Most POD cases were observed on the first day after surgery [32.5% (13 of 40) in the preparation group vs. 9.8% (4 of 41) in the non-preparation group, *P* = 0.025]. The median [IQR (range)] severity of POD, expressed as the highest CAM-S scores, were similar between the preparation group and non-preparation group {4.0 [3.0–4.5 (3.0–5.0)] vs. 4.0 [3.3–4.8 (3.0–5.0)] points, *P* = 0.97}. Furthermore, there was no significant difference in POD duration between the preparation group and non-preparation group {2.0 [1.5–3.0 (1.0–4.0)] vs. 2.0 [1.0–3.0 (1.0–3.0)] days, *P* = 0.786}.

According to our result, the POD patients were 13 in the preparation group and 4 in the non-preparation group, so a total of 17 patients were classified into the POD group. The non-POD group patients were 27 in the preparation group and 37 in the non-preparation group, so a total of 64 patients in the non-POD group. Demographic data, anesthetic and surgical data, and postoperative data in the POD group and the non-POD group were documented and *P*-values were analyzed in Supplementary Table 1. The factors “age, duration of surgery, and mechanical bowel preparation” were significantly different in POD group and non-POD group (*P* = 0.025, *P* = 0.009, and *P* = 0.025, respectively). A similar result was acquired in the univariate analysis. We found that age [risk ratio, 1.16; 95% confidence interval (CI), 1.01–1.32; *P* = 0.032], duration of surgery (risk ratio, 1.01; 95% CI, 1.00–1.02; *P* = 0.019), and mechanical bowel preparation (risk ratio, 4.45; 95% CI, 1.31–15.17; *P* = 0.017) were significantly associated with delirium in univariate analysis. In multivariate analyses, when combining age and duration of surgery in multivariate analyses, we found that patients with MBP had a 4.792-fold higher odds of POD than those without MBP (CI: 1.274–18.028; *P* = 0.020) (Table 4).

**TABLE 4:** Univariable and multivariate logistic regression analysis of the factors associated with postoperative delirium.

Variables	Univariable			Multivariable		
	Unadjusted OR	95%CI	*P*-value	Adjusted OR	95%CI	*P*-value
Age	1.155	1.012, 1.317	0.032	1.200	1.030, 1.398	0.019
ASA physical status III	2.071	0.460, 9.319	0.343			
Preoperative baseline MMSE scores	0.820	0.540, 1.245	0.351			
Duration of surgery	1.010	1.002, 1.018	0.019	1.010	1.001, 1.019	0.036
Duration of anesthesia	1.006	0.999, 1.014	0.107			
EBL	1.000	0.995, 1.005	0.953			
Mechanical bowel preparation	4.454	1.308, 15.169	0.017	4.792	1.274, 18.028	0.020

*MMSE, Mini-Mental State Examination; EBL, estimated blood loss; OR, odds ratio.*

### Alternation of Gut Microbiota Between Preparation Group and Non-preparation Group

First, we explored the α-diversity and β-diversity of the gut microbiota in the preparation group and the non-preparation group before mechanical bowel preparation.

A -diversity refers to the diversity of bacteria or species within a community or habitat. It is mainly concerned with the number of bacteria or species (Bermon et al., 2015). B -diversity refers to the alternation rate of bacteria or species composition between different habitats along the environment gradient; it is also known as between-habitat diversity (Nakov et al., 2015).

**FIGURE 3:**
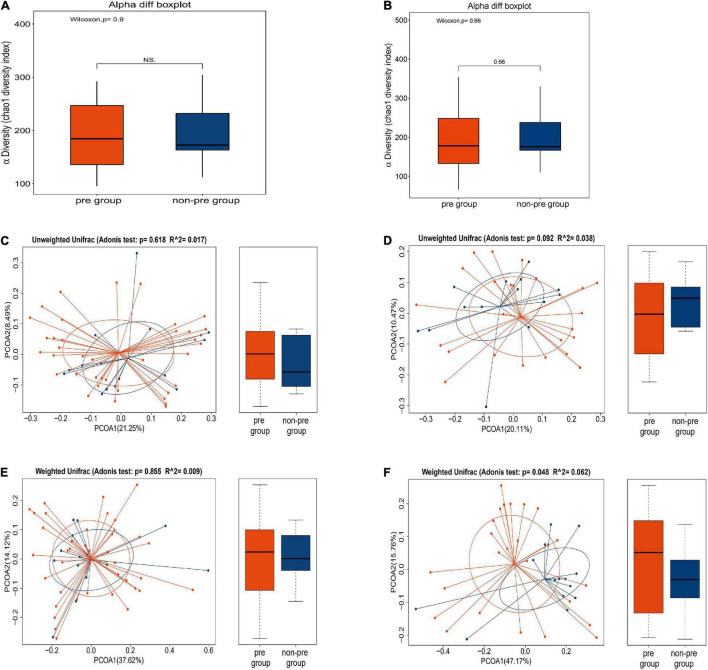
**(A,C,E)** Different profiles of the gut microbiota before MBP between the preparation group and non-preparation group. **(A)** α-diversity: box plots showed differences in the fecal microbiome diversity indices between the two groups according to the Chao 1 index based on the OTU counts. **(C)** β-diversity: outcome of Adonis tests with Unweighted UniFracs distances for the two groups. **(E)** β-diversity: outcome of Adonis tests with Weighted UniFracs distances for the two groups. The yellow plot showed data of the preparation group, and the blue plot showed data of the non-preparation group. **(B,D,F)** Different profiles of the gut microbiota after MBP between the preparation group and non-preparation group. **(B)** α-diversity: box plots showed differences in the fecal microbiome diversity indices between the two groups according to the Chao 1 index based on the OTU counts. **(D)** β-diversity: outcome of Adonis tests with Unweighted UniFracs distances for the two groups. **(F)** β-diversity: outcome of Adonis tests with Weighted UniFracs distances for the two groups. The yellow plot showed data of the preparation group, and the blue plot showed data of the non-preparation group.

We found that there was no difference in both α-diversity (Figure 3A) (*P* = 0.9) and β-diversity (Figures 3C,E) between the preparation group and non-preparation group before MBP (Adonis *R*^2^ = 0.017, *P* = 0.618, based on the unweighted data; Adonis *R*^2^ = 0.009, *P* = 0.855, based on the weighted). It indicated that the basic composition of gut microbiota between the preparation group and the non-preparation group was similar.

Then, we further investigated α-diversity and β-diversity of the gut microbiota in the preparation group and the non-preparation group after mechanical bowel preparation.

We found that there was no change in α-diversity between samples after MBP from 2 groups (*P* = 0.66) (Figure 3B). However, difference was found in β-diversity (Figures 3D,F) between the preparation group and the non-preparation group after MBP (Adonis *R*^2^ = 0.038, *P* = 0.092, based on unweighted data; Adonis *R*^2^ = 0.062, *P* = 0.048, based on the weighted). Taken together, we could infer that MBP did change the composition and state of gut microbiota.

### Abundance of the Composition of Gut Microbiota in the Preparation Group and Non-preparation Group After Mechanical Bowel Preparation

**FIGURE 4:**
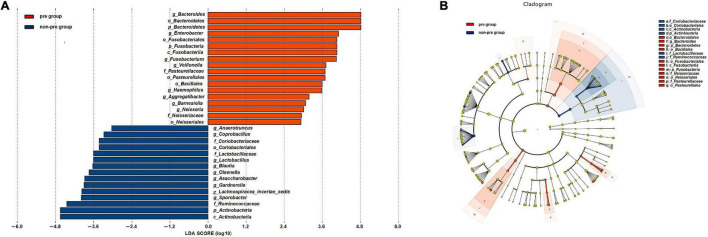
**(A)** Changes in the gut microbiota after mechanical bowel preparation. The histogram of the LDA scores presented the relative abundance of the main bacterial in the two groups in the fecal samples. Blue boxes referred to the bacteria contributing a lot in the non-preparation groups, yellow boxes referred to the bacteria contributing a lot in the preparation group. **(B)** LEfSe results in a cladogram on fecal microbiomes between the two groups. Taxonomic representation of statistically and biologically consistent differences between the two groups. Each circle’s diameter was proportional to the taxon’s abundance. Blue nodes referred to the bacteria contributing a lot in the non-preparation groups, yellow nodes referred to the bacteria contributing a lot in the preparation group.

We conducted a supervised comparison on the microbiota between the preparation group and the non-preparation group by utilizing the LEfSe analysis. We used a logarithmic LDA score with a cutoff value of 2.0 to identify important taxonomic differences between the preparation group and non-preparation group after MBP (Figures 4A,B). Our results suggested there was a remarkable difference in fecal microbiota between the two groups. We found that at the phylum level, the abundance of *Bacteroidetes* and *Fusobacteria* were obviously higher in the preparation group (*P* < 0.05), while *Actinobacteria* was more abundant in the non-preparation group. At the order level, *Pasteurellales* and *Bacillales* were obviously higher in the preparation group, while *Coriobacteriales* was higher in the non-preparation group. At the family level, *Pasteurellaceae* and *Neisseriaceae* were higher in the preparation group, while *Lactobacillaceae* and *Ruminococcaceae* were obviously higher in the non-preparation group. At the genus level, *Bacteroides, Enterobacter, Fusobacterium*, *Veillonella, Haemophilus*, *Aggreatibacter*, *Barnesiella*, and *Neisseria* were significantly higher in the preparation group (*P* < 0.05), while a relatively higher abundance of *Anaerotruncus, Coprobacillus, Lactobacillus, Blautia*, *Olsenella*, *Asaccharobacter*, *Gardnerella*, *Lachnospiracea_incertae_sedis*, and *Sporobacter* were in the non-preparation group (Figures 4A,B).

### Alternations in the Taxa Between Postoperative Delirium and Non-postoperative Delirium Groups

**FIGURE 5:**
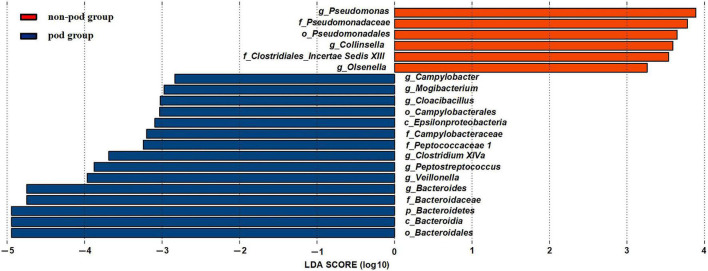
Changes in the gut microbiota between the POD group and non-POD group. The histogram of the LDA scores presented the relative abundance of the main bacterial in the two groups in the fecal samples. Blue boxes referred to the bacteria contributing a lot in the POD groups, yellow boxes referred to the bacteria contributing a lot in the non-POD group.

To further investigate the correlation between gut microbiota and the incidence of POD, we examined the different microbiota between the second fecal samples of all POD patients (the total number is 17) and all the non-POD patients (the total number is 64) by the LEfSe analysis as we assumed it was this fecal status that caused POD in patients. We used a logarithmic LDA score cutoff of 2.0 to identify important taxonomic differences between the POD group and the non-POD group. Our results suggested a remarkable difference in fecal microbiota between the POD and non-POD groups. We found that the abundance of genus *Bacteroides*, *Mogibacterium, Campylobacter, Cloacibacillus*, *Clostridium XIVa*, *Peptostreptococcus*, and *Veillonella* were obviously higher in the POD group (*P* < 0.05), while the abundance of genus *Pseudomonas*, *Collinsella*, and *Olsenella* were significantly higher in the non-POD group (Figure 5).

Among them, *Bacteroides* genus and *Veillonella* genus significantly increased after MBP. Thus, we suspected that they might be the crime bacteria causing POD caused by MBP.

*Olsenella* genus was relatively higher in the non-preparation group which was also higher in the non-POD group. Thus we suspected that it might be a beneficial bacteria to reduce the incidence of POD.

## Discussion

According to our result, we found that mechanical bowel preparation could increase the incidence of POD in patients who underwent radical gastrectomy. Also, we found that the alternation of the gut microbiota caused by MBP, especially the abundance of *Bacteroides* and *Veillonella*, might be a risk factor of POD.

Mechanical bowel preparation (MBP) for elective gastric surgery has long been regarded as a clinical routine for many decades. Recently, it is reported that MBP introduces alterations to the intestinal microbiota. However, the results are not identical. In our study, we found that bowel preparation introduces significant alternations to the intestinal microbiota in terms of β-diversity (based on the weighted, Adonis *R*^2^ = 0.062, *P* = 0.048). We found that the abundance of genus *Bacteroidetes*, *Enterobacter, Fusobacterium*, *Veillonella, Haemophilus*, *Aggreatibacter*, *Barnesiella*, and *Neisseria* were obviously higher in the preparation group, while genus *Anaerotruncus, Coprobacillus, Lactobacillus, Blautia*, *Olsenella*, *Asaccharobacter*, *Gardnerella*, *Lachnospiracea_incertae-sedis*, and *Sporobacter* were in the non-preparation group (*P* < 0.05).

It was easy to understand some taxa of microbiota were decreased after MBP. However, there are several taxa of microbiota elevated after MBP. We speculated these bacteria could replicate more freely when some other bacteria were decreased by MBP.

More importantly, we found that MBP is a risk factor of POD (32.5% vs. 9.8%, *P* = 0.025). The multivariate analysis revealed that patients with MBP had a 4.792-fold higher odds of POD than those without MBP (CI: 1.274–18.028; *P* = 0.020). Apart from MBP, delirium was also associated with patients’ age and the duration of surgery according to our results, which were consistent with previous findings (Fukata et al., 2017). Our result showed that other factors such as ASA physical status III and intraoperative hypotension were not associated with POD, as other studies (Marcantonio et al., 1998). Wang et al. (2015) has found that intraoperative blood loss of less than 300 ml would not contribute to POD. As only five patients whose blood loss was greater than 300 ml. So we did not find it as a risk factor for POD. Other factors might cause POD, like cognitive impairment would interfere with the incidence of POD (Fukata et al., 2017). However, as a prospective study, we created inclusion criteria for patients, like that the preoperative baseline MMSE scores of all the patients selected in our study were higher than 24. So we did not find factors like preoperative baseline MMSE scores as a risk factor for POD. According to a previous study, anticholinergic drugs are commonly associated with cognitive changes, namely, hallucinations, and overt delirium (Molchan et al., 1992). So anticholinergic drugs were replaced by other cardiovascular active drugs such as isoprotereno in our study when the heart rate fell. Masatsugu Hiraki has found that maximum intraoperative temperature ≥ 37°C is an independent risk factor of early postoperative delirium after laparoscopic colorectal surgery in elderly patients (Hiraki et al., 2021). So we strictly controlled and monitored the patients’ temperature at 36.3–36.9°C.

Next, we analyzed the difference in gut microbiota between the POD groups and non-POD groups. Among them, the abundance of genus *Bacteroides* and *Veillonella* were higher in the MBP group than the non-MBP group when we compared the second fecal samples between the groups. Thus, we considered the increased abundance of *Bacteroides* and *Veillonella* after MBP might be a factor causing POD in patients scheduled for radical gastrectomy in this study.

A previous study has found that *Bacteroides* could impair cognitive function (Cattaneo et al., 2017). Saji et al. (2019) have found that an increased prevalence of *Bacteroides* is independently associated with the presence of mild cognitive impairment. Apart from these, some other studies revealed that patients with Alzheimer’s disease have a larger population of *Bacterodietes* in their gut (Guo et al., 2021). These results could support our study that *Bacteroides* might be a risk factor for POD.

Moreover, a previous study has also revealed that the genus *Veillonella* (Liu et al., 2021) which belongs to the phylum Firmicutes, contributed to cognitive dysfunction. Liu et al. (2021) have found that *Veillonellacea* was negatively correlated with orientation and delayed in patients with mild cognitive impairment. V*eillonellaceae* was also associated with the severity of schizophrenia (Zheng et al., 2019). According to our result, the genus *Veillonella* might also play a role in causing POD.

We also found that the genus *Pseudomonas*, *Collinsella*, and *Olsenella* were relatively higher in the non-POD group. So, these genera could be assumed to have protective effects on cognitive function.

The relative abundance of *Olsenella* was also higher in the non-preparation group, further indicating its role in preventing POD. It has been reported that most of *Olsenella* could produce acetic acid, one part of SCFAs (Zhang et al., 2020). The SCFAs (mainly acetate, propionate, and butyrate) exert crucial physiological effects against several cognitive dysfunction, namely, depression, Alzheimer’s disease (AD), and Parkinson’s disease (PD), and autism spectrum disorder (ASD) (Dalile et al., 2019). Furthermore, SCFA administration has been proposed as a treatment target for such cognitive dysfunction (Dalile et al., 2019). So, we speculated that *Olsenella* might help reduce the incidence of POD by producing SCFAs.

*Pseudomonas genus* has been found to do good to the nervous system. It was reported that halophilic crude (extracts from *Pseudomonas zhaodongensis*) exerted protective effects against memory deficits and anxiety- and depression-like behaviors in methionine-induced schizophrenia in mice (Massaoudi et al., 2020), which was consistent with our results.

However, the role of *Collinsella* on the nervous system was not consistent. *Collinsella* was found to be increased in patients with AD but decreased in relapsing-remitting multiple sclerosis which is a disease also presenting cognitive dysfunction (Ling et al., 2020). Another study found that diet introduced *Collinsella* increasing did not interfere with cognition (van Soest et al., 2020). Further study is needed to validate its function in cognition as many factors such as different disease patterns, sequencing techniques, and geographical location might be responsible for the different roles of *Collinsella*.

As for other bacteria altered after MBP, although we did not find a correlation between them and POD, they are still worth studying. Among them, the genus *Lactobacillus*, family *Ruminococcaceae* were significantly decreased after MBP. Many studies have shown that they are beneficial to the central nervous system. Wen et al. (2020) showed that *Lactobacillus* protected the postoperative cognitive functions of the aged mice with gut dysbiosis. It could also prevent the learning and memory deficits induced by anesthesia/surgery (Jiang et al., 2019). Another study has found that probiotic consumption (containing *Lactobacillus*) for 12 weeks improved cognitive function in patients with AD (Akbari et al., 2016).

Furthermore, Zhang et al. (2019) have found that POD mice had fewer *Ruminococcaceae* compared with the non-POD ones in their digestive tract. Ren et al. (2020) have found that patients with PD who present cognitive impairment had a lower abundance of genus *Ruminococcus* in their fecal samples. With more patients enrolled, we might identify more exact taxa of gut microbiota contribute to POD or cognitive dysfunction.

Our study does have limitations. One limitation of this study was the relatively small number of patients enrolled. Further studies are needed to detect the effect of MBP on a greater number of individuals; another limitation was that our study mainly focused on the instant effect of changes in the microbiota composition on patients’ cognitive function. We will continue to monitor a relatively long time of patients’ cognitive status to detect the long-term impact of MBP on patients’ cognitive function and gut microbiota.

## Conclusion

In conclusion, we found that, compared with the non-preparation group, mechanical bowel preparation increased the incidence of postoperative delirium in the patient with gastric cancer. Among all the genera altered by the MBP, genus *Bacteroides* and genus *Veillonella*, which were increased in the POD group, might participate in the pathogenesis of POD. Meanwhile, genus *Olsenella* which was relatively higher in both the non-preparation and non-POD groups might reduce the incidence of POD.

## Data Availability Statement

The datasets presented in this study can be found in online repositories. The names of the repository/repositories and accession number(s) can be found below: 10.6084/m9.figshare.18095891.

## Ethics Statement

The studies involving human participants were reviewed and approved by the ethical committee of Huashan Hospital (approval number: KY2018-354). The patients/participants provided their written informed consent to participate in this study. Written informed consent was obtained from the individual(s) for the publication of any potentially identifiable images or data included in this article.

## Author Contributions

YW designed the study, revised the manuscript, and participated in the final approval of the version to be submitted. ZY was involved in writing the manuscript and interpreting the data. CT collected the data and analyzed the data. XQ collected the data and completed figures and tables. HW revised the manuscript. All authors reviewed and approved the final version of the manuscript.

## Conflict of Interest

The authors declare that the research was conducted in the absence of any commercial or financial relationships that could be construed as a potential conflict of interest.

## Publisher’s Note

All claims expressed in this article are solely those of the authors and do not necessarily represent those of their affiliated organizations, or those of the publisher, the editors and the reviewers. Any product that may be evaluated in this article, or claim that may be made by its manufacturer, is not guaranteed or endorsed by the publisher.

## Abbreviations

MBP: mechanical bowel preparation
pre group: preparation group
non-pre group: non-preparation group
POD: postoperative delirium.
