# Supplementary material for: Mechanical Bowel Preparation Is a Risk Factor for Postoperative Delirium as It Alters the Gut Microbiota Composition: A Prospective Randomized Single-Center Study
Source: Front Aging Neurosci. 2022 Apr 4;14:847610. doi: 10.3389/fnagi.2022.847610 (PMC9014128; doi:10.3389/fnagi.2022.847610)
Supplement: Supplementary file 1 [file Table_1.docx]

**Supplement Table 1.**Demographic Data, Anesthetic and Surgical Data and Postoperative Data in the POD group and non-POD group. Data were expressed as mean (SD),median (IQR[range]) or number (Proportion).

|  | **POD group**  **(n=17)** | **Non-POD group**  **(n=64)** | **P value** |
| --- | --- | --- | --- |
| Male sex; n | 10 (58.8%) | 37 (57.8%) | 0.940 |
| Age;yr | 76 (5) | 73 (4) | 0.025 |
| BMI; kg/m^2^ | 23.1 (2.4) | 23.9 (3.2) | 0.352 |
| Education |  |  |  |
| primary school education | 9 (53%) | 28 (44%) | 0.544 |
| secondary school education | 8 (47%) | 30 (47%) |  |
| university education | 0 (0%) | 6 (9%) |  |
| Heavy drinker*^1^ |  |  |  |
| yes | 4 (24%) | 18 (28%) | 0.943 |
| no | 13 (76%) | 46 (72%) |  |
| Current smoker |  |  |  |
| yes | 5 (29.4%) | 15 (23.4%) | 0.848 |
| no | 12 (70.6%) | 49 (76.6%) |  |
| ASA physical status |  |  |  |
| Ⅰ | 7 (41.2%) | 24 (37.5%) | 0.534 |
| Ⅱ | 7 (41.2%) | 34 (53.1%) |  |
| Ⅲ | 3 (17.6%) | 6 (9.4%) |  |
| Preoperative baseline MMSE scores (0-30) | 27.2 (1.1) | 27.6 (1.4) | 0.392 |
| Preoperative HAMD scores | 2.4 (2.3) | 2.9 (2.3) | 0.429 |
| Hemoglobin |  |  |  |
| normal | 14 (82%) | 50 (78%) | 0.964 |
| abnormal | 3 (18%) | 14 (22%) |  |
| Tumor stage |  |  |  |
| T1N0M0 | 7 (41%) | 20 (31%) | 0.440 |
| T2N0M0 | 10 (59%) | 44 (69%) |  |
| diabetes |  |  |  |
|  |  |  |  |
| Yes | 6 (35.3%) | 16 (25.0%) | 0.396 |
| No | 11 (64.7%) | 48 (75.0%) |  |
| nutritional impairment*^2^ |  |  |  |
| Yes | 1 (6%) | 2 (3%) | 0.512 |
| No | 16 (94% | 62 (97%) |  |
| functional dependency*^3^ |  |  |  |
| Yes | 0 (0.0%) | 0 (0%) | 0.999 |
| No | 17 (100%) | 64 (100%) |  |
| Duration of surgery ;min | 230 (180-300 [151-407]) | 188 (154-225 [95-436]) | 0.009 |
| Duration of anesthesia; min | 250 (213-350 [190-466]) | 253 (206-280 [130-480] | 0.240 |
| EBL;ml | 150 (100-200 [100-400]) | 150 (100-200  [40-600]) | 0.862 |
| Pain NRS score (0-10) |  |  |  |
| 24h | 3 (3-6 [2-8]) | 3 (3-4 [0-9]) | 0.265 |
| 48h | 3 (2-3 [0-5]) | 3 (2-3 [0-5]) | 0.957 |
| 72h | 1 (1-2 [0-3]) | 1 (0-3 [0-3]) | 0.778 |
| Cumulative rescue morphine consumption;mg | 5.0 (4.0-5.0 [2.0-10.0]) | 4.5 (3.0-5.8 [0.0-8.0]) | 0.507 |
| Postoperative time out of bed; days | 4.0 (2.0-4.0  [1.0-6.0]) | 3.0 (2.0-4.0 [1.0-7.0]) | 0.318 |
| Length of hospital stay; days | 8.0 (5.0-10.0 [3.0-17.0]) | 9.0 (7.0-10.0 [5.0-24.0]) | 0.159 |
| Mechancial bower preparation |  |  |  |
| Yes | 13 | 27 | 0.025 |
| No | 4 | 37 |  |

BMI, body mass index; MMSE: Mini-Mental State Examination; HAMD,Hamilton depression scale; pre group,preparation group;non-pre group,non-prepaaton group;EBL, estimated blood loss;NRS, numerical rating scale;*1 Defined as current intake of alcohol, on average, 3-4drinks per day at least four times per week;*2 Defined as BMI <18.5 kg/m2;*3 Defined as Functional Activites Questionnaire（FAQ）score ≥5 .
